# Localized sclerostin accumulation in osteocyte lacunar-canalicular system is associated with cortical bone microstructural alterations and bone fragility in *db/db* male mice

**DOI:** 10.3389/fcell.2025.1562764

**Published:** 2025-04-25

**Authors:** Xingfan Wu, Yuanli Ai, Yutao He, Dan Ma, Xi Li, Xiao Huang, Rui Cheng, Bin Wang

**Affiliations:** ^1^ Obesity and Metabolic Diseases Research Center, Department of Physiology, College of Basic Medicine, Chongqing Medical University, Chongqing, China; ^2^ Hunan Provincial Key Laboratory of Dong Medicine, Biomedical Research Institute, Hunan University of Medicine, Huaihua, China; ^3^ Liupanshui People’s Hospital, Liupanshui, Guizhou, China

**Keywords:** sclerostin, lacunar-canalicular system, diabetes, bone fragility, localized accumulation

## Abstract

Bone fragility in type 2 diabetes mellitus (T2DM) is often characterized by impaired bone quality, despite normal or increased bone mineral density. Serum sclerostin levels are elevated in diabetes, yet its role in bone fragility is not fully understood. Sclerostin (SOST) is a Wnt signaling inhibitor primarily secreted by osteocytes, regulating bone formation and homeostasis. Sclerostin inhibits Wnt signaling, suppressing osteoblast differentiation and activity, which limits bone formation. However, the localized effects of sclerostin within the osteocyte lacunar-canalicular system (LCS) and its contribution to bone fragility in T2DM remain unclear. In this study, we investigated the role of elevated sclerostin in bone fragility using the *db/db* mice. We found that *db/db* mice exhibited significant osteoporosis, increased bone fragility, and structural damage to the LCS. Sclerostin expression was elevated, and its accumulation within the cortical bone LCS correlated with increased expression of matrix-degrading enzymes, including Cathepsin K (CTSK) and matrix metalloproteinase 13 (MMP-13). Further *in vitro* experiments with recombinant sclerostin confirmed the upregulation of these enzymes, suggesting that sclerostin’s local effects within the LCS contribute to matrix degradation. These preliminary findings indicate that localized sclerostin accumulation in LCS is associated with cortical bone microstructural alterations and fragility in *db/db* male mice. This study highlights the potential of targeting sclerostin’s local effects within the LCS as a therapeutic strategy to prevent bone deterioration in diabetes.

## 1 Introduction

Type 2 diabetes mellitus (T2DM) is the predominant type of diabetes and a growing global health concern. Despite normal or even increased bone mineral density (BMD), T2DM patients have a higher fracture risk, a clinical paradox, as higher BMD typically indicates greater bone strength (Moayeri et al., 2017; Nilsson et al., 2017). This paradox suggests that bone strength depends not only on BMD but also on bone quality, which determines the structural and material integrity of bone. Bone quality encompasses microarchitecture, material properties, and cellular function (Alliston, 2014; Fonseca et al., 2014; Morgan et al., 2018; Vahidi et al., 2021). In T2DM, metabolic disturbances (e.g., insulin resistance, hyperglycemia, and chronic inflammation) disrupt bone remodeling, thereby compromising bone quality and elevating fracture risk (Khosla et al., 2021). Clarifying the influence of these T2DM-related abnormalities on bone quality is essential for understanding the pathogenesis of diabetic bone fragility.

Osteocytes, embedded within the lacunar-canalicular system (LCS), play a central role in bone remodeling and quality maintenance (Bonewald, 2011). Sclerostin (Sost), a key regulatory protein secreted by osteocytes, inhibits Wnt signaling pathway by binding to LRP5/6, suppressing bone formation (Marini et al., 2023). Beyond its systemic effects, sclerostin accumulates within the LCS under mechanical unloading (Osumi et al., 2021). Sclerostin is thought to regulate local processes within the LCS. It upregulates matrix-degrading enzymes including carbonic anhydrase 2 (CA2), cathepsin K (CTSK), and tartrate-resistant acid phosphatase (ACP5), promoting osteocyte-driven pericellular matrix resorption (Kogawa et al., 2013), a process known as perilacunar/canalicular remodeling (PLR) (Heveran and Boerckel, 2023). However, the localized effects of sclerostin on LCS integrity and its contribution to bone fragility, particularly in the context of T2DM, remain unclear.

In T2DM, elevated sclerostin levels have been reported in both serum and bone tissue, but their contribution to bone fragility is debated (Pacicca et al., 2019; Sabancilar et al., 2023). Pereira et al. reported that elevated sclerostin does not correlate with increased osteocyte apoptosis or empty lacuna formation (Pereira et al., 2016), whereas others highlight its potential role in impairing LCS integrity. For instance, sclerostin has been shown to regulate lacuna size via the LRP4/5/6 pathway (Kogawa et al., 2013; Kogawa et al., 2018), while alterations in TGF-β and parathyroid hormone (PTH) signaling have been associated with disrupted LCS remodeling in T2DM (Gardinier et al., 2016; Picke et al., 2019). These findings indicate that sclerostin may contribute to bone fragility in T2DM by modulating the osteocyte microenvironment, but direct evidence is lacking.

This study investigates the role of localized sclerostin accumulation in diabetic bone fragility using the *db/db* mouse model. We hypothesize that increased sclerostin accumulation in osteocyte LCS exerts *in situ* effects, contributes to microstructural damage and osteocyte dysfunction, ultimately leading to increased bone fragility. Additionally, we used the IDG-SW3 cell model to initially assess the impact of high glucose and recombinant sclerostin on osteocyte remodeling markers, thereby exploring how sclerostin modulates osteocyte behavior in the local microenvironment. By focusing on the interplay among sclerostin, PLR-associated enzymes, and the LCS microenvironment, this study aims to provide new insights into the mechanisms underlying T2DM-related bone quality deterioration and explore potential therapeutic targets for fracture prevention in diabetic patients.

## 2 Materials and methods

### 2.1 Animal model and specimen preparation

Male *db/db* mice (BKS-Lepr^em2Cd479^/Gpt, 8 weeks old; n = 5) and age- and sex-matched wild-type (WT) controls (C57BLKS/JGpt background; n = 5) were obtained from GemPharmatech Co., Ltd. Animals were housed in a pathogen-free, temperature-controlled environment with a 12-h light/dark cycle and had free access to standard chow and water for 12 weeks. Before sacrifice, body weight and fasting blood glucose levels were recorded. Blood, pancreatic islets, and bone samples were then collected for analysis. At euthanasia, blood was collected from the orbital sinus using capillary tubes under aseptic conditions. The blood was allowed to clot at room temperature for 30 min, then cooled and centrifuged at 3,000 g for 15 min to separate the serum, which was subsequently aliquoted and stored at −80°C until analysis.

To confirm the successful induction of T2DM in our *db/db* mouse model, pancreatic islets were collected and islet damage was assessed using immunofluorescence staining. Pancreas samples were fixed in 4% paraformaldehyde, embedded in paraffin, and sectioned at 7 µm. After deparaffinization, rehydration, and antigen retrieval with citrate buffer, sections were blocked with 5% BSA and incubated overnight at 4°C with an insulin antibody (4590S, Cell Signaling Technology, United States; 1:100). After a 60-min incubation with Anti-rabbit IgG (H + L), F (ab')_2_ Fragment (Alexa Fluor® 488 Conjugate) (4412S, Cell Signaling Technology, United States; 1:1,000) at room temperature and 10-min DAPI staining (D9542, Sigma-Aldrich, United States; 1:500), images were captured using an Olympus BX43 fluorescence microscope.

For microCT and three-point bending tests, left femurs were dissected, wrapped in PBS-soaked gauze, and stored at −20°C until microCT imaging. Right femurs were fixed in 4% paraformaldehyde for 48 h for histological analysis. For TEM, a 1-mm section ∼3 mm from the proximal end of the left tibia was excised and fixed. The remaining left tibia was preserved for microindentation by wrapping in PBS-soaked gauze and freezing at −20°C. All other bones were snap-frozen in liquid nitrogen for future use.

All procedures were approved by the Institutional Animal Care and Use Committee (IACUC) of Chongqing Medical University and conducted in accordance with relevant guidelines.

### 2.2 Micro-CT assessment

Micro-computed tomography (Micro-CT) was performed using a SkyScan 1,276 system (Bruker). Left femurs were scanned with a voxel size of 6 μm, using an X-ray source set at 55 kV and 200 μA with a 0.25 mm aluminum filter. Images were acquired at a 0.3° rotation step and an exposure time of 517 ms. Reconstruction of the projection data was conducted with SkyScan NRecon software (Version 1.7.4.2). Micro-CT grayscale thresholding was applied for image segmentation. For trabecular bone, a threshold range of 65–255 was used (corresponding approximately to 1,328–8133 HU). For cortical bone, the threshold range was 100–255 (approximately 2,581–8133 HU). Trabecular bone parameters were evaluated in a volume of interest (VOI) comprising a 1.8-mm-thick section, starting 500 μm proximal to the growth plate of the distal femur. Bone parameters included: trabecular bone volume fraction (BV/TV, %), trabecular number (Tb.N, 1/mm), trabecular separation (Tb.Sp, mm), trabecular thickness (Tb.Th, mm), structure model index (SMI), and connectivity density (Conn.D, 1/mm^3^). For cortical bone assessment, a VOI was defined at the midshaft over a 0.6-mm-thick section. Parameters measured included cortical thickness (Ct.Th, mm), cortical bone area (Ct.Ar, mm^2^).

### 2.3 Three-point bending test

To determine the whole-bone mechanical properties, after microCT scanning, the left femurs were subjected to three-point bending tests to failure. The mechanical tests were conducted using a mechanical testing system (CellScale, Canada) with a 6 mm support span and a loading rate of 0.05 mm/s. Load-displacement data were collected at 0.2-s intervals. Stiffness, maximum load, post-yield displacement (PYD), and work to fracture were derived from the recorded force-displacement data. The elastic modulus (E, GPa) was calculated using the formula Equation 1 (Jepsen et al., 2015):

$$E=\frac{{KL}^{3}}{48I_{\min}}$$ (1)

where *K* is the stiffness, *L* is the loading span, *I*_min_ is the minimum moment of inertia.

### 2.4 Microindentation

The elastic modulus of the tibial mid-diaphyseal cortical bone was determined on its transverse section using microindentation. Preserved samples were thawed to room temperature, and the tibial mid-diaphyseal cortical bone was sectioned horizontally. The distal tibia was sequentially polished using 1,200-, 2,500-, and 4000-grit sandpapers, then further refined with 3 μm and 1 μm diamond suspensions and 0.05 CR polishing paste, followed by thorough rinsing with ddH_2_O. To remove any residual debris from polishing, the samples were subsequently sonicated in deionized water for 5 min. Following sonication, a 1 mm diamond indenter was pressed into the cortical bone cross sectional surface using a micro-Vickers hardness tester (MHVS-1000BZ, China). The test was performed with a peak load of 0.05 N and a 10-s dwell time at maximum load. At least 20 indentations per sample were evenly distributed to ensure measurement accuracy. Indentation hardness (Hv) values were measured directly from individual indent profiles at ×400 magnification, and the young’s modulus (Ei) was calculated using the formula (Currey and Brear, 1990): Ei = 0.36Hv + 0.58 (*R* ^2^ = 93%).

### 2.5 Transmission electron microscopy

A 1-mm mid-tibial segment was fixed in a TEM-specific fixative solution (4% paraformaldehyde, 2% glutaraldehyde, and 0.7% ruthenium trichloride in 0.05 M sodium dicarboxylic acid sodium arsenate) for 48 h, followed by decalcification at 4°C for 21 days. After decalcification, samples were rinsed with 0.1 M PBS, post-fixed in 1% osmium tetroxide for 2 h, and sequentially dehydrated in graded ethanol and acetone before epoxy resin infiltration and polymerization. Ultrathin sections (50 nm) were cut with a Reichert ultramicrotome, stained with a saturated solution of 4.0% uranyl acetate and 0.4% lead citrate, and examined using a JEOL JEM-1400Plus TEM at 80 kV. High-resolution images were acquired using a Morada G2 CCD camera at ×12,000 magnification for osteocyte lacunae and ×50,000 for canaliculi. LCS ultrastructure was analyzed by manually tracing these features in Adobe Photoshop and quantifying dimensions with the BoneJ plugin in Fiji.

### 2.6 Bone histomorphometry

After fixation in 4% paraformaldehyde, decalcification, and ethanol dehydration, the right femurs were embedded in paraffin. Eight-micrometer thick coronal sections of the distal femur were prepared. After deparaffinization, rehydration, and rinsing, the sections were stained using H&E, TRAP, silver nitrate, and immunohistochemistry. Parameters including osteoblast number (N.Ob/BS), osteoblast surface (Ob.S/BS), osteoclast number (N.Oc/BS), and osteoclast surface (Oc.S/BS) were measured using ImageJ based on H&E and TRAP staining. Canalicular density and lacunar area were analyzed via silver nitrate staining to evaluate osteocyte network integrity.

### 2.7 Immunohistochemistry

Paraffin-embedded femoral sections were stained CTSK (DF6614, Affinity, China), MMP-13 (AF5355, Affinity, China), and Sclerostin (AF1589, R&D Systems, United States) following standard protocols. After deparaffinization and rehydration, antigen retrieval was performed with digestive enzymes (AR0022, Boster, China) at 37°C for 30 min. CTSK and MMP-13 were detected using the SPlink Detection Kit (SAP-9000, ZSGB-Bio, China), based on a mouse/rabbit streptavidin–biotin–HRP system. Sclerostin was detected using the Goat Two-step Detection Kit (PV-9003, ZSGB-Bio, China), which employs a polymer-conjugated HRP system. Staining was visualized using DAB condensed chromogen (PR30010, Proteintech, China). Images were acquired using an Olympus BX43 microscope with ×20/0.40 NA and ×40/0.65 NA objectives. DAB-positive cells were quantified from ×20 images, and representative images were captured at ×40.

### 2.8 Biochemistry tests

Prior to analysis, the frozen serum samples were thawed at room temperature. Serum concentrations of the bone resorption markers collagen C-terminal telopeptide (CTX-1; E03C0235, Blugene, China) and tartrate-resistant acid phosphatase (TRAcP 5b; QZ-24680, Jiubang, China), as well as the bone formation marker osteocalcin (E03O0001, Blugene, China) and sclerostin (Sost; QZ-23778, Jiubang, China), were measured using commercially available ELISA kits following the manufacturer’s instructions.

### 2.9 IDG-SW3 cell culture and treatments

IDG-SW3 cells were cultured in αMEM supplemented with 10% fetal bovine serum (FBS), 1% penicillin/streptomycin (P/S), and 50 U/mL IFN-γ on rat tail type I collagen-coated plates at 33°C with 5% CO_2_ for cell expansion. For osteogenic differentiation, cells were seeded at a density of 80,000 cells/cm^2^ on rat tail type I collagen-coated plates under osteogenic conditions (without IFN-γ) at 37°C in the presence of 50 μg/mL ascorbic acid and 4 mM β-glycerophosphate. After 28 days of induction, one set of cells were fixed with 4% PFA and then stained with the ALP staining kit (C3250S, Beyotime, China) to assess early osteogenic activity, and with the ARS staining kit (C0148S, Beyotime, China) to evaluate matrix mineralization. In separate experiments, for high glucose treatment, live cells (without prior fixation) maintained in differentiation medium after 28 days of osteogenic induction were supplemented with D-glucose to a final concentration of 20 mM for 2 days, while control groups were maintained at a normal glucose concentration of 5.5 mM. For recombinant sclerostin (SCL) treatment, IDG-SW3 cells were treated with 100 ng/mL recombinant mouse sclerostin (HY-P70717, MCE, China) for 48 h following 28 days of osteogenic induction.

### 2.10 Quantitative real-time polymerase chain reaction

Total RNA was extracted from IDG-SW3 cells using TRIzol reagent (#349902, Invitrogen, United States) and quantified spectrophotometrically. mRNA was reverse-transcribed into cDNA using a cDNA synthesis kit (K16225, Thermo Fisher Scientific, United States). cDNA amplification was performed using SYBR Green Master Mix (A25742, Thermo Fisher Scientific, United States) and specific primer sequences listed in Table 1, following the manufacturer’s instructions. A QuantStudio 3 or 5 Real-Time PCR System (Thermo Fisher Scientific, United States) was used for amplification. Data were analyzed using the 2^−ΔΔCT^ method with *Gapdh* as the reference gene for normalization.

**TABLE 1:** Primers used for quantitative real-time PCR.

Gene Name	Primer
*Gapdh*	Foward:5′-AGGTCGGTGTGAACGGATTTG-3′ Reverse:5′-TGTAGACCATGTAGTTGAGGTCA-3′
*Ctsk*	Foward:5′-AATACCTCCCTCTCGATCCTACA-3′ Reverse:5′-TGGTTCTTGACTGGAGTAACGTA-3′
*Mmp13*	Foward:5′-CTTCTTCTTGTTGAGCTGGACTC-3′ Reverse:5′-CTGTGGAGGTCACTGTAGACT-3′
*Atp6v0d2*	Foward:5′-TCTTGAGTTTGAGGCCGACAG-3′
	Reverse:5′-GCAACCCCTCTGGATAGAGC-3′
*Fgf23*	Foward:5′-TGCAAACGCTCGAACTCTCT-3′
	Reverse:5′-GCTGCCATTTTGGGGTTAGC-3′
*Mepe*	Foward:5′-GTGCTGCCCTCCTCAGAAAT-3′
	Reverse:5′-GAGCTTTCAGGACCAGACCC-3′
*Pdpn*	Foward:5′-GGACCGTGCCAGTGTTGTTCTG-3′
	Reverse:5′-ACCATGCCGTCTCCTGTACCTG-3′
*Phex*	Foward:5′-TCATTGATACCAGACTCTACC-3′
	Reverse:5′-CAATGGTTTTCTTCCTCTCG-3′
*Sost*	Foward:5′-CGTGCCTCATCTGCCTACTTGTG-3′
	Reverse:5′-CCGGTTCATGGTCTGGTTGTTCTC-3′

### 2.11 Statistical analysis

Statistical analyses were performed using GraphPad Prism (GraphPad Software). An unpaired Student’s t-test was used for group comparisons. Pearson correlation analysis and linear regression analysis were conducted to evaluate the relationships between the proportions of sclerostin-positive cells and CTSK- and MMP-13-positive cells, based on immunohistochemistry (IHC) data. Results are presented as mean ± SD. Statistical significance was defined as p < 0.05, with thresholds specified as follows: *p < 0.05, **p < 0.01, ***p < 0.001, and ****p < 0.0001.

## 3 Result

### 3.1 Reduced bone mass and bone turnover in 20-week-old male *db/db* mice

**FIGURE 1:**
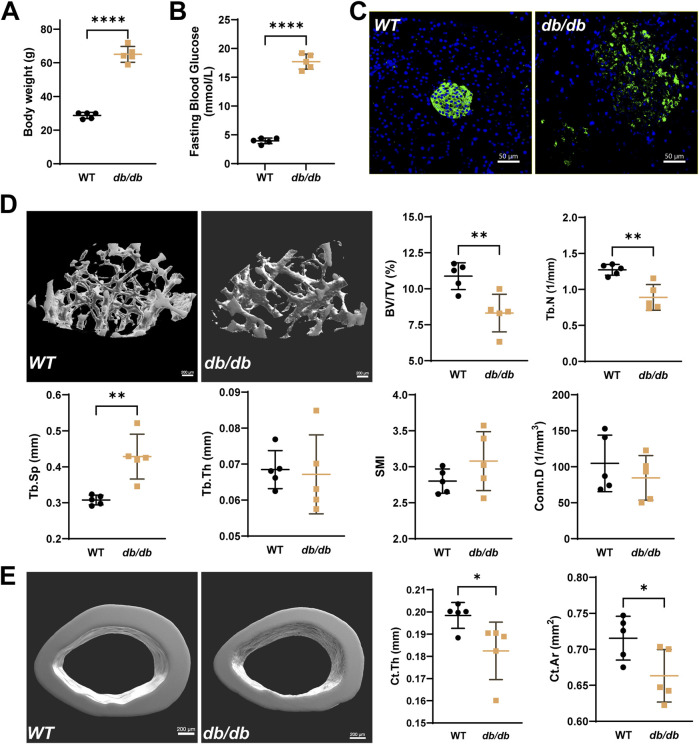
Reduced bone microstructure in 20-week-old male *db/db* diabetic mice. Compared to WT, *db/db* mice show significantly higher body weight **(A)** and fasting serum glucose levels **(B)**, accompanied by disrupted islet structures **(C)**, typical of T2DM (Green: insulin; Blue: DAPI; scale bar = 50 μm). **(D)** Representative micro-CT images of the distal femur metaphysis show reduced BV/TV and Tb.N, increased Tb.Sp, and minor changes in Tb.Th, SMI, and Conn.D in *db/db* mice. **(E)** Micro-CT analysis of the femoral midshaft shows significantly lower Ct.Th and Ct.Ar in *db/db* mice. Data are presented as mean ± SD, and analyzed by unpaired Student’s t-test (n = 5 mice per group). *p < 0.05, **p < 0.01, ****P < 0.0001.

In 20-week-old male *db/db* mice, typical T2DM phenotypes were observed, including significantly higher body weight and fasting blood glucose levels compared to WT mice (Figures 1A,B). Immunofluorescence staining revealed disrupted pancreatic islet structures, characterized by disorganized insulin distribution (Figure 1C). Micro-CT analysis showed significant changes in bone microstructure in *db/db* femurs. Compared to the wild-type group, the distal femur metaphyseal trabecular bone in *db/db* mice exhibited reduced BV/TV and Tb.N, increased Tb.Sp, and slight changes in Tb.Th, SMI, and Conn.D (Figure 1D). The midshaft cortical bone showed a significant reduction in Ct.Th and Ct.Ar. (Figure 1E).

**FIGURE 2:**
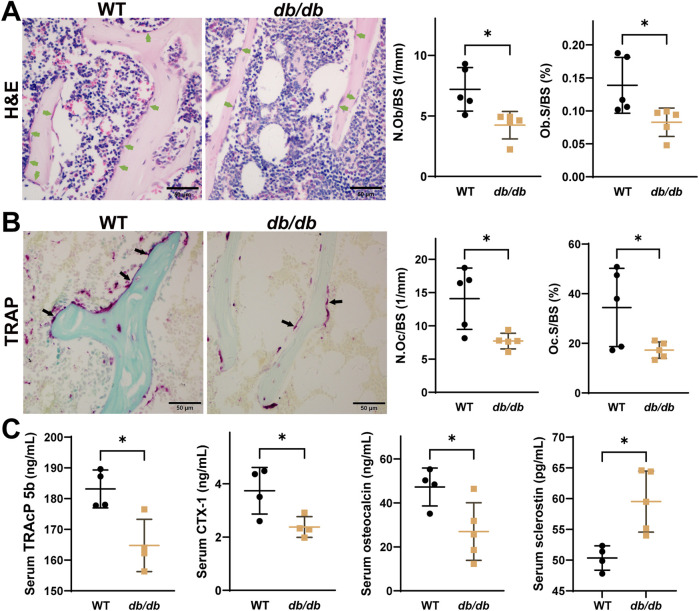
Reduced osteoblast and osteoclast activity in 20-week-old male *db/db* diabetic mice. H&E **(A)** and TRAP **(B)** staining show reduced activity of both osteoblasts and osteoclasts in *db/db* mice (n = 5 per group). **(C)** Serum analysis shows lower osteocalcin, TRACP 5b, and CTX-1 levels, and higher sclerostin levels in *db/db* mice (n = 4-5 per group). Data are presented as mean ± SD, analyzed by unpaired Student’s t-test. *p < 0.05, **p < 0.01.

H&E staining indicated decreased osteoblast numbers (N.Ob/BS) and osteoblast surface area (Ob.S/BS) (Figure 2A), while TRAP staining showed reductions in osteoclast numbers (N.Oc/BS) and surface area (Oc.S/BS) (Figure 2B). Serum analysis revealed lower levels of osteocalcin, TRAcP 5b, and CTX-1, along with higher levels of sclerostin in *db/db* mice compared to WT mice (Figure 2C).

### 3.2 Bone mechanical properties impaired in 20-week-old male *db/db* mice

**FIGURE 3:**
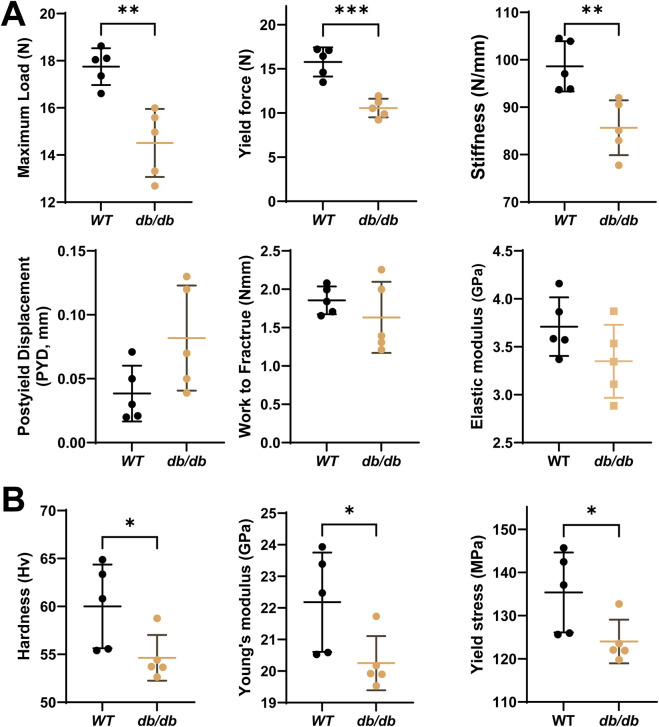
Decreased bone mechanical properties in 20-week-old male *db/db* diabetic mice cortical bone. **(A)** The 3-point bending test on left femurs shows significantly reduced maximum load (N), stiffness (N/mm), yield force (N) and slightly increased post-yield displacement (PYD, mm), with a minor reduction in work to fracture (Nm) and elastic modulus (E, Gpa) in *db/db* mice compared to WT. **(B)** The micro-indentation test on the mid-diaphysis of the tibia reveals significantly lower hardness (Hv), Young’s modulus (Ei) and yield stress in *db/db* mice compared to WT. Data are presented as mean ± SD. Statistical analysis was performed using an unpaired Student’s t-test (n = 5 mice per group). *p < 0.05, **p < 0.01.

Mechanical testing revealed impaired cortical bone properties in *db/db* mice. The three-point bending test, performed on the left femurs, showed reductions in maximum load, stiffness, and yield force. Post-yield displacement was slightly increased, while work to fracture and elastic modulus showed no significant differences compared to WT mice (Figure 3A). These results suggest that cortical bone in *db/db* mice is weaker and less stiff, with reduced strength but similar fracture energy.

Microindentation testing, conducted on the mid-diaphyseal cross-section of the left tibia, demonstrated significantly lower hardness, Young’s modulus, and yield stress in *db/db* mice compared to WT controls (Figure 3B). Reduced hardness may indicate poor mineralization and altered bone matrix. Lower yield stress suggests the bone fails at lower stress levels, increasing fracture risk.

### 3.3 Altered osteocyte LCS in 20-week-old male *db/db* mice cortical bone

**FIGURE 4:**
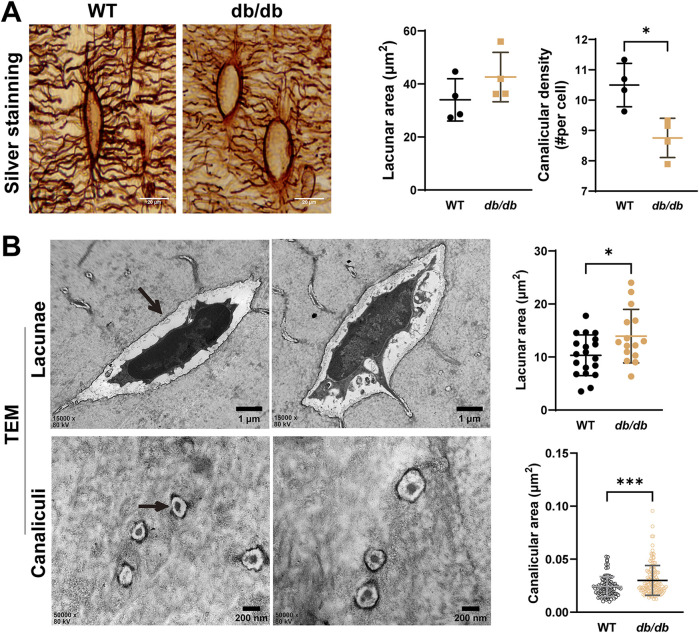
Altered osteocyte lacunar-canalicular system (LCS) in 20-week-old male *db/db* diabetic mice cortical bone. **(A)** silver nitrate staining of LCS showing reduced lacunae and canaliculi in *db/db* mice compared to WT mice (n = 4 mice per group). **(B)** Representative TEM images of lacunae (n = 15–20) and canaliculi (n = 96–142) in cross-section reveal structural changes in the tibiae of *db/db* mice compared to WT mice. Data are presented as mean ± SD. Statistical analysis was performed using a two-tailed unpaired Student’s t-test. *p < 0.05, ***p < 0.001.

To examine the microstructural changes in the osteocyte LCS of cortical bone in *db/db* mice, photon silver staining was performed. Statistical analysis showed that the lacunar area in the cortical bone of *db/db* mice was slightly larger than in WT mice but did not reach statistical significance. However, the canalicular density per osteocyte was significantly reduced in *db/db* mice compared to WT controls (Figure 4A).

Transmission electron microscopy (TEM) was used to analyze the ultrastructure of the LCS in cortical bone. TEM results revealed significantly larger lacunae and reduced canalicular area in *db/db* mice compared to WT mice, indicating ultrastructural disruptions in both lacunae and canaliculi during the progression of T2DM (Figure 4B).

### 3.4 Sclerostin and PLR-related enzyme expression in 20-week-old male *db/db* mice cortical bone

**FIGURE 5:**
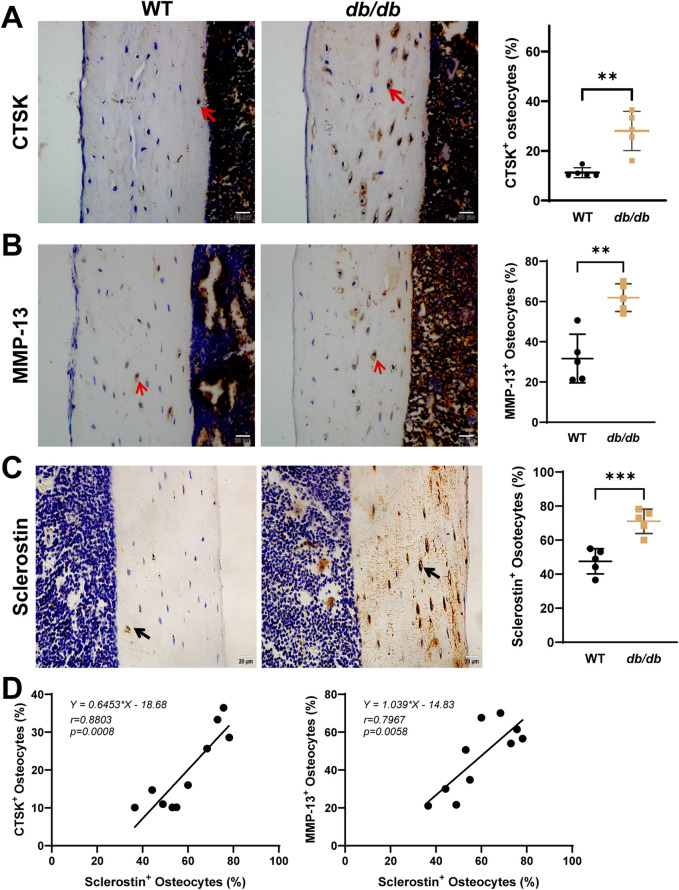
Localized sclerostin accumulation and increased expression of PLR-related proteins in 20-week-old male *db/db* diabetic mice cortical bone. Immunohistochemical staining shows increased expression of CTSK **(A)**, MMP-13 **(B)**, and sclerostin **(C)** within the osteocyte LCS of *db/db* mice compared to WT mice. Linear regression analysis **(D)** reveals a significant positive correlation between sclerostin expression and CTSK and MMP-13 using combined data from both groups. Data are presented as mean ± SD. Statistical analysis was performed using an unpaired Student’s t-test for **(A–C)** (n = 5 mice per group) and Pearson correlation for **(D)**. *p < 0.05, **p < 0.01, ***p < 0.001.

Immunohistochemistry revealed significantly increased osteocyte positivity for the PLR-related enzymes CTSK and MMP-13 in *db/db* mice compared to WT controls (Figures 5A,B). The expression of these enzymes appeared more pronounced across the lacuna in *db/db* mice. Sclerostin expression was significantly elevated in db/db mice and appeared more concentrated within the lacuna (Figure 5C). Pearson correlation analysis demonstrated a significant positive correlation between sclerostin expression and CTSK (*r* = 0.8803, *p* = 0.0008) as well as MMP-13 (*r* = 0.7967, *p* = 0.0058), highlighting a potential association between sclerostin and PLR-related enzyme activity in *db/db* mice (Figure 5D).

### 3.5 Changes in osteocyte PLR-related enzymes under exogenous sclerostin and high-glucose conditions

**FIGURE 6:**
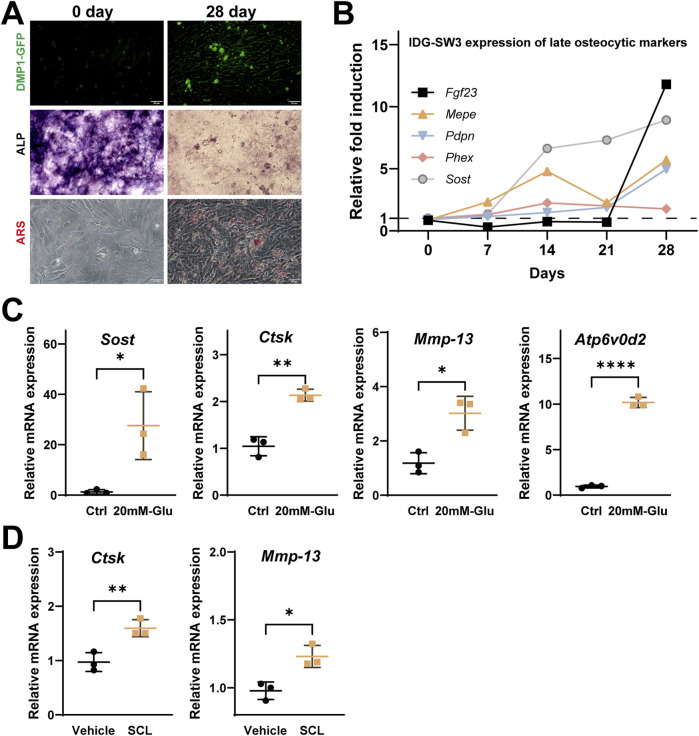
Effects of high glucose and recombinant Sclerostin (SCL) on osteocyte remodeling markers in IDG-SW3 cells. **(A)** Representative images showing osteocytic differentiation over 28 days, including DMP1-GFP fluorescence, ALP staining, and ARS staining. **(B)** Time-course expression of late osteocytic markers (FGF23, MEPE, Pdpn, Phex, and Sost) over 28 days, presented as relative fold induction. **(C)** Elevated mRNA expression of *Sost, Ctsk, Mmp-13, and Atp6v0d2* in IDG-SW3 cells treated with 20 mM glucose compared to control cells. **(D)** Upregulation of *Ctsk and Mmp-13* mRNA levels in IDG-SW3 cells treated with recombinant SCL compared to vehicle control. Data represent mean ± SD from three independent experiments (n = 3). Statistical significance is indicated (*p < 0.05, **p < 0.01, ****p < 0.0001).

To investigate the effects of sclerostin on osteocyte PLR-related enzyme expression, IDG-SW3 cells were differentiated for 28 days to mimic mature osteocytes (Figures 6A,B). High-glucose conditions (20 mM) were used to simulate the diabetic environment in *db/db* mice, while recombinant mouse sclerostin protein (SCL, 100 ng/mL) was added to simulate the localized sclerostin accumulation observed around osteocytes.

Under high-glucose conditions, qPCR analysis showed significantly elevated mRNA levels of *Sost, Ctsk, Mmp-13, and Atp6v0d2* compared to the control group (Figure 6C). Similarly, treatment with exogenous SCL significantly upregulated the expression of *Ctsk and Mmp-13* in 28-day differentiated IDG-SW3 cells, highlighting the role of elevated sclerostin levels in influencing osteocyte PLR-related enzyme expression (Figure 6D).

## 4 Discussion

The objective of this study is to preliminarily explore the role of sclerostin in the osteocyte LCS and its localized contribution to diabetic bone fragility. Using the *db/db* mouse model, we demonstrated that elevated SOST levels and disrupted LCS architecture may play significant roles in impairing bone quality in T2DM. By promoting PLR related enzyme expression and inducing matrix degradation, SOST accumulation within the LCS appears to directly contribute to local skeletal fragility.

Consistent with previous findings, *db/db* mice exhibited reduced trabecular and cortical bone mass (Qiu et al., 2024), accompanied by impaired mechanical properties such as reduced maximum load and stiffness. These results align with the hypothesis that hyperglycemia and insulin resistance compromise bone quality by disrupting bone remodeling processes (Imerb et al., 2020; Dardari and Segurens, 2024). Gao et al. indicated that AGEs-induced nanoscale alterations in collagen may contribute to skeletal fragility in T2DM (Gao et al., 2024).

Data from the three-point bending test on the femur indicated a trend toward reduced elastic modulus in *db/db* mice, although this difference did not reach statistical significance. In contrast, microindentation testing on the tibia mid-shaft revealed a statistically significant reduction in Young’s modulus. These differences likely stem from the inherent distinctions between the two methods. Three-point bending evaluates the overall structural response of the bone by integrating factors such as geometry, cross-sectional moment of inertia, and material distribution, which may mask localized impairments. Conversely, microindentation directly measures tissue-level mechanical properties and is more sensitive to changes in the bone matrix, such as decreased mineralization and altered collagen integrity. Although anatomical differences between the femur and tibia might also play a role, our findings primarily support the notion that local changes in bone quality are key contributors to diabetic bone fragility.

The LCS is central to osteocyte mechanosensation and bone homeostasis (Bonewald, 2011). Our results revealed significant disruptions in the LCS of *db/db* mice, including enlarged lacunae, widened canaliculi, and reduced canalicular density. These changes may impair the fluid flow and mechanical strain transmission necessary for osteocyte function (Sang and Ural, 2023). Importantly, immunohistochemical analysis demonstrated elevated SOST expression and its redistribution within the LCS, with accumulation extending into the canaliculi. This localized increase in SOST may directly impair PLR, consistent with previous studies linking sclerostin to disrupted bone quality in metabolic conditions (Moustafa et al., 2012; Osumi et al., 2021).

*In vitro* experiments using IDG-SW3 cells under high-glucose conditions and recombinant sclerostin (SCL) treatment provided additional evidence for the role of SOST in PLR dysregulation. High glucose elevated SOST expression along with PLR-related enzymes such as CTSK and MMP-13, simulating the diabetic bone microenvironment (Kogawa et al., 2013; Pacicca et al., 2019). Similarly, exogenous SCL induced the expression of these matrix-degrading enzymes, highlighting the dual impact of hyperglycemia and sclerostin on osteocyte-driven matrix remodeling.

The localized accumulation of SOST within the LCS may result from mechanical unloading, AGE accumulation, or impaired proteasomal degradation (Sakamoto et al., 2019; Gould et al., 2021; Osumi et al., 2021). Elevated SOST likely contributes to LCS dysfunction through two primary mechanisms: upregulation of PLR-related matrix-degrading enzymes and inhibition of the Wnt/β-catenin pathway. These disruptions exacerbate LCS structural damage and impair bone matrix integrity, potentially increasing skeletal fragility in diabetes. Therapeutically, targeting SOST with neutralizing antibodies has shown promise in improving bone mass and quality (Marini et al., 2023). However, its site-specific effects within the LCS and interactions with PLR-related pathways warrant further investigation.

There are several limitations to this study, although it provides preliminary insights into the role of SOST and LCS in diabetic bone fragility. The use of a single animal model limits the generalizability of our findings to human conditions. Moreover, the precise mechanisms of SOST accumulation and its regulation of PLR-related pathways require further elucidation. Future studies should focus on the interplay between SOST, PLR-related enzymes, and the diabetic microenvironment to identify targeted interventions for mitigating bone fragility.

In conclusion, our findings suggest that elevated SOST levels and disrupted LCS architecture are likely central to the impaired bone quality in *db/db* mice. By promoting PLR-related enzyme expression, SOST appears to contribute to localized LCS dysfunction and matrix degradation, ultimately leading to increased bone fragility. These results highlight the LCS and SOST as potential therapeutic targets for addressing skeletal complications in diabetes.

## Data Availability

The original contributions presented in the study are included in the article/supplementary material, further inquiries can be directed to the corresponding authors.
